# Report on the First WEGECA Meeting: Women Endoscopists for Global Exchange and Career Advancement

**DOI:** 10.1111/den.70105

**Published:** 2026-01-20

**Authors:** Mayo Tanabe, Reiko Ashida, Akiko Shiotani, Naomi Kakushima, Haruhiro Inoue, Shinji Tanaka

**Affiliations:** ^1^ Digestive Diseases Center Showa Medical University Koto Toyosu Hospital Tokyo Japan; ^2^ Second Department of Internal Medicine Wakayama Medical University Wakayama Japan; ^3^ Department of Gastroenterology Kawasaki Medical University Okayama Japan; ^4^ Department of Gastroenterology Graduate School of Medicine, The University of Tokyo Bunkyo‐ku Japan; ^5^ JA Onomichi General Hospital Onomichi Japan

**Keywords:** gender diversity, international collaboration, leadership, mentorship, networking

## 1 Introduction

The inaugural WEGECA (Women Endoscopists for Global Exchange and Career Advancement) assembly was held on September 6, 2025, at the Toshi Center Hotel in Tokyo, following the first JGES International main program. This historic event marked the official launch of a new collaborative initiative under the Japanese Gastroenterological Endoscopy Society (JGES), dedicated to promoting gender diversity, leadership, and international collaboration among women in endoscopy.

WEGECA operates as part of the Career Support Committee for Female Endoscopists, which aims to foster mentorship, equity, and career advancement across generations and to strengthen international exchange and collaboration among women in endoscopy. Supported by the JGES Board of Directors and the International Committee, this first meeting symbolized a transformative moment for gender inclusion within Japan's endoscopic community and for global engagement in the field.

## 2 Opening Remarks and Institutional Support

The session began with warm words from Dr. Naomi Kakushima, Vice Chair of the Career Support Committee for Female Endoscopists, who spoke on behalf of Chair Prof. Akiko Shiotani. She highlighted the committee's long‐standing efforts to establish regional branches nationwide and expressed appreciation for the participants who gathered to celebrate this new milestone. Dr. Kakushima emphasized that WEGECA embodies the shared aspiration to empower women, cultivate mentorship, and expand professional networks that transcend institutional and national boundaries.

A special address by Prof. Shinji Tanaka, President of JGES, reaffirmed the Society's commitment to diversity and equity. He commended the establishment of WEGECA as a necessary and forward‐looking initiative that reflects the evolving global landscape of endoscopy. His presence—along with that of other senior leaders—conferred strong institutional endorsement and reinforced the importance of integrating gender equality into the Society's long‐term vision.

## 3 Invited Lectures and Discussions

**FIGURE 1:**
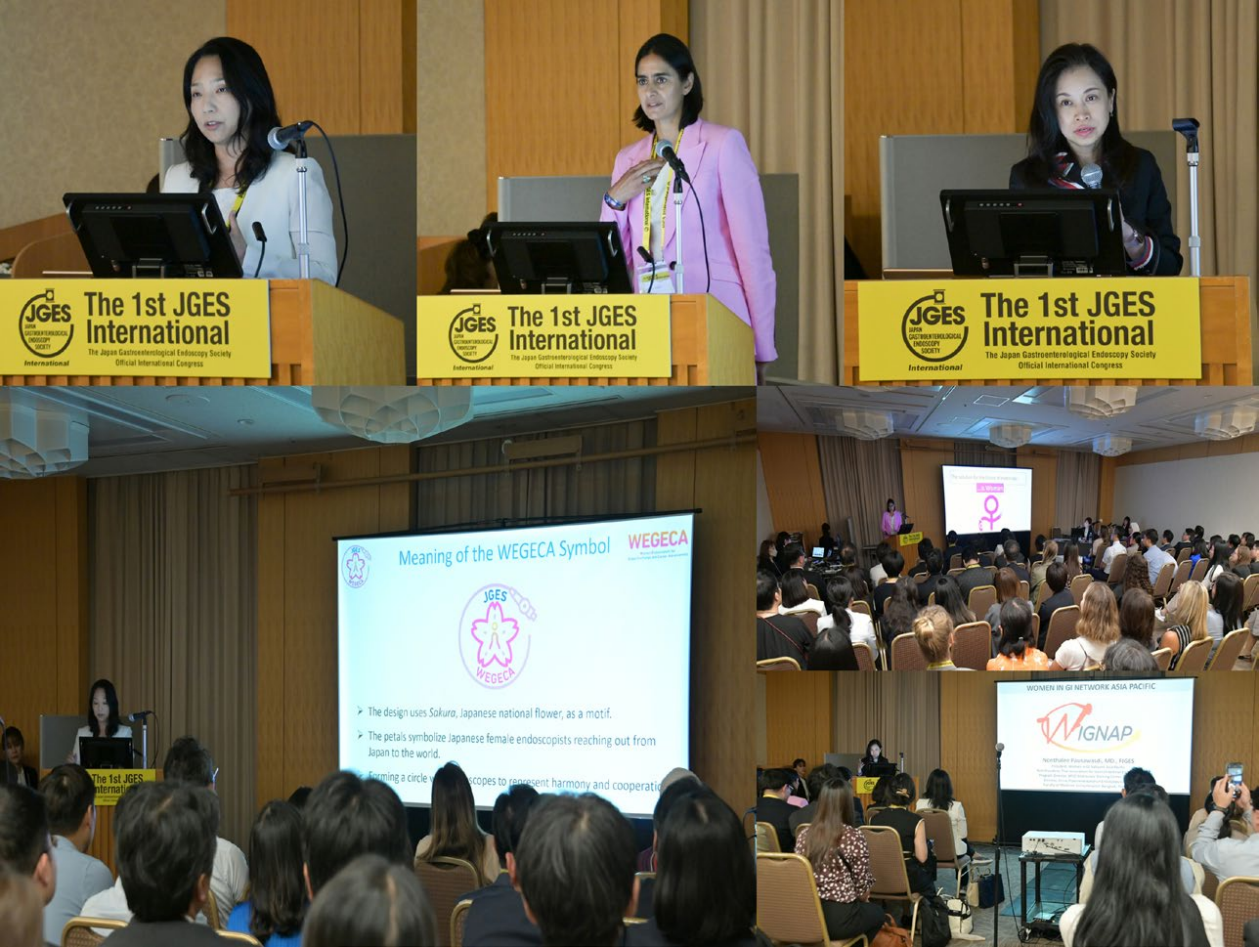
Representative scene from the first WEGECA Assembly, showing invited speakers during the meeting (from left to right: Dr. Reiko Ashida [WEGECA], Dr. Amrita Sethi [WIE], and Dr. Nonthalee Pausawasdi [WIGNAP]).

The invited lectures highlighted the complementary roles of three organizations dedicated to advancing gender equity and international collaboration in endoscopy: WEGECA in Japan, Women in Endoscopy (WIE), a global organization headquartered in the United States, and the Women in Gastroenterology Network Asia Pacific (WIGNAP). Together, these organizations represent regionally rooted yet globally connected networks that share common goals while addressing distinct local and regional needs (Figure 1).

## 4 The Vision of WEGECA: Dr. Reiko Ashida

Dr. Reiko Ashida (Chair of WEGECA; Wakayama Medical University) delivered the first invited lecture at the inaugural WEGECA Assembly. She outlined the current landscape of female endoscopists in Japan and explained how WEGECA was founded in response to the need for structured mentorship, greater visibility, and international collaboration. Dr. Ashida also highlighted the symbolism of the WEGECA logo—representing harmony, unity, and empowerment—and described the committee's mission to foster leadership and global connectivity among women in endoscopy. Drawing on her clinical and educational experience, she emphasized that gender diversity is not merely an issue of equity but a driver of innovation and excellence in endoscopic medicine. Her lecture captured the essence of WEGECA—collaboration, inclusivity, and shared progress—toward a more connected and sustainable future in endoscopy.

## 5 Women in Endoscopy (WIE): Dr. Amrita Sethi

Representing Women in Endoscopy (WIE), Prof. Amrita Sethi (Columbia University, USA) congratulated WEGECA on its establishment and praised the remarkable turnout at its first assembly. She emphasized that WIE's purpose is not simply to empower women, but to create an environment where “everyone who loves endoscopy can do what they want to do.” Dr. Sethi discussed key challenges faced by women—limited leadership opportunities, implicit bias, imposter syndrome, and concerns related to family, fertility, and radiation exposure—and outlined WIE's strategies to address them through mentorship, role modeling, and international collaboration. She summarized the organization's guiding principles as four pillars: Network, Platform, Ally, and Mentor, noting that true progress depends on the interconnection of these elements. In closing, she stressed that WIE's mission is not for women alone but for men and women together to advance endoscopy through inclusion and shared responsibility, a message that deeply resonated with the audience and aligned with WEGECA's vision of global collaboration.

## 6 Women in Gastroenterology Network Asia Pacific (WIGNAP): Dr. Nonthalee Pausawasdi

Prof. Nonthalee Pausawasdi (Mahidol University, Thailand) introduced the background and current activities of the Women in Gastroenterology Network Asia Pacific (WIGNAP), which connects female gastroenterologists across the region to promote education, research, and leadership development. She described WIGNAP's key initiatives—regional workshops, mentorship programs, and leadership forums—aimed at strengthening collaboration and professional growth among women physicians in Asia. Presenting comparative data, Prof. Pausawasdi noted that while women's representation in gastroenterology continues to rise in many Asian countries, Japan still has a relatively smaller proportion of female endoscopists. She expressed optimism that WEGECA will help enhance regional engagement and mutual support, further advancing equity and innovation across the Asia–Pacific community.

## 7 The Synergy of Global and Regional Networks

These three organizations represent a multi‐layered support system for women in the field. While WEGECA focuses on the specific needs of endoscopists in Japan, it operates in close synergy with WIGNAP, which serves the broader Asia–Pacific region, and WIE, which provides a global platform from its U.S. base. This collaborative framework ensures that mentorship and professional growth are fostered both within local contexts and across international borders, reinforcing a sustainable and inclusive network for women in endoscopy.

## 8 Networking and Community Building

**FIGURE 2:**
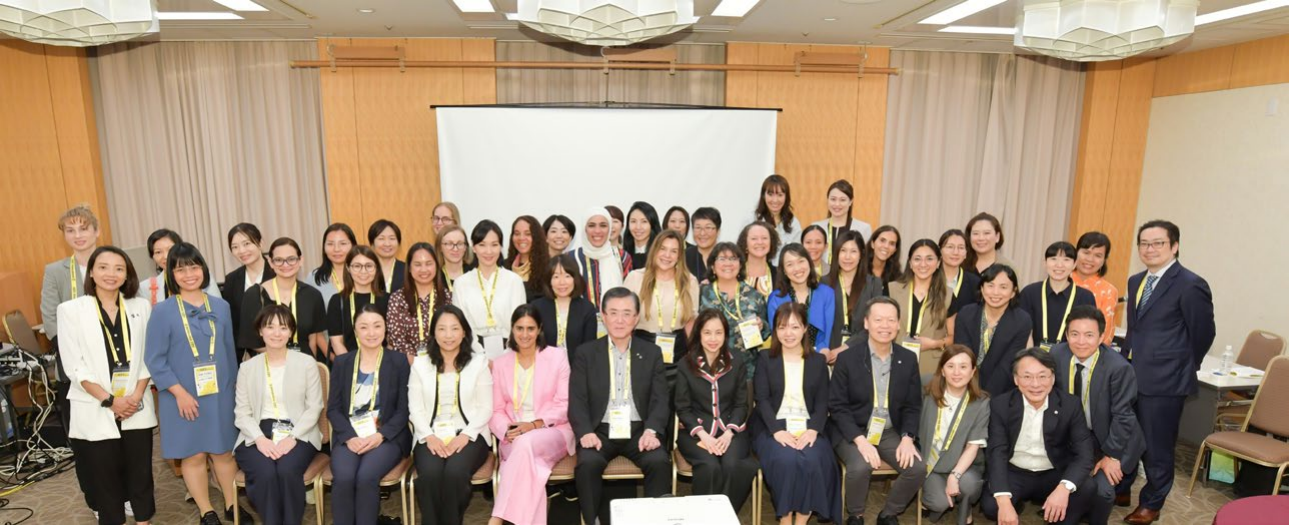
Group photograph taken after the first WEGECA Assembly, held following the first JGES International Meeting. Participants included speakers, moderators, and attendees from Japan and abroad, who gathered for an open and friendly exchange.

Following the formal lectures, a networking session provided an open and dynamic platform for discussion among participants from Japan and abroad. Physicians from various generations and institutions—together with supportive male colleagues—shared their experiences on mentorship, leadership, and balancing professional and personal lives. The atmosphere was warm, engaging, and inclusive, characterized by genuine mutual encouragement. Many young female doctors remarked that meeting senior leaders in an informal setting was deeply motivating, while others appreciated that the event's inclusive tone—emphasizing partnership rather than segregation—represented a meaningful step forward for the Japanese endoscopic community. The session also helped strengthen connections among Japanese endoscopists themselves, fostering new collaborations and friendships across institutions. The evening concluded with a celebratory toast, leaving participants with a sense of optimism and camaraderie, and symbolizing the shared hope that WEGECA will continue to flourish as a platform for learning, collaboration, and international exchange (Figure 2).

## 9 Outcomes and Impact

The inaugural WEGECA meeting produced outcomes that far surpassed expectations. It successfully established a cross‐border network linking Japanese endoscopists with international communities such as WIE and WIGNAP, fostering ideas for future joint programs and mentoring exchanges. By aligning with JGES International, the event gained strong visibility and institutional credibility, reflecting Japan's growing commitment to gender inclusion in endoscopy. Importantly, the meeting demonstrated that gender equity in medicine can advance most effectively through structured, academically grounded collaboration rather than advocacy alone. The shared enthusiasm and diversity of participants signified a cultural shift toward a more inclusive professional environment—one where leadership is defined by talent and contribution, not by gender.

## 10 Challenges and Future Directions

As with any inaugural event, several areas for improvement were identified. The transition between lectures and networking sessions revealed the need for more refined time management and smoother coordination to sustain audience engagement. Feedback also emphasized the importance of inclusivity. Although WEGECA centers on women's advancement, future meetings will encourage participation from all genders—reflecting the philosophy of WIE and WIGNAP—to foster collaboration that transcends gender boundaries. Looking ahead, the WEGECA Committee plans to adopt more interactive formats such as mentorship roundtables, panel discussions, and small‐group dialogues. The committee also aims to integrate WEGECA more formally into the JGES program, strengthening its institutional presence and ensuring its sustainability as a recurring platform for diversity and professional development. The WEGECA Assembly will continue to be organized regularly, serving as a lasting forum for education, mentorship, and international collaboration under JGES.

## 11 Conclusion

The first WEGECA Assembly marked a defining moment in advancing gender diversity and international collaboration within Japanese endoscopy. The enthusiasm, leadership, and solidarity demonstrated by participants reflected a shared determination to build a more equitable and globally connected future. Through the collaboration of JGES, WIE, and WIGNAP, WEGECA has laid a strong foundation for sustained mentorship, research, and international exchange. Its success illustrates that gender equity is not a peripheral agenda but a core component of excellence and progress in modern endoscopic medicine. Moving forward, WEGECA will continue to expand global partnerships, nurture emerging leaders, and promote innovation grounded in diversity, empathy, and shared vision—further strengthening a globally connected and inclusive endoscopic community.

## Funding

The authors have nothing to report.

## Conflicts of Interest

The authors declare no conflicts of interest.

